# ADAMTS13 as a thromboinflammatory brake at the vascular-immune interface: from viral immunothrombosis to the tumor microenvironment

**DOI:** 10.3389/fimmu.2026.1922929

**Published:** 2026-09-18

**Authors:** Peng Huang, Xiaoyu Sun, Jie Peng, Wei Tian

**Affiliations:** 1 Department of Interventional Oncology, Zibo Central Hospital, Zibo, Shandong, China; 2 Department of Pathology, Zibo Central Hospital, Zibo, Shandong, China; 3 Department of Oncology, Zibo Central Hospital, Zibo, Shandong, China

**Keywords:** ADAMTS13, cancer-associated thrombosis, complement, immune checkpoint inhibitor, immunothrombosis, thromboinflammation, tumor microenvironment, von Willebrand factor

## Abstract

ADAMTS13 is conventionally regarded as the protease whose severe deficiency causes thrombotic thrombocytopenic purpura (TTP). By cleaving shear-unfolded ultra-large and high-molecular-weight von Willebrand factor (VWF), ADAMTS13 limits a platelet- and leukocyte-adhesive vascular scaffold. We propose a narrower and testable extension of this biology to cancer: an imbalance between VWF burden and ADAMTS13 processing capacity may act as a context-dependent modifier or amplifier of thromboinflammation in selected VWF-rich, shear-exposed tumor vascular niches, but is not established as an initiating or universally rate-limiting cause of cancer-associated thrombosis. We define “relative ADAMTS13 insufficiency” as a research phenotype rather than a diagnosis or evidence of causality. It refers to ADAMTS13 activity outside the severe-deficiency range, typically ≥10%, together with increased VWF burden and a disproportionately low ADAMTS13 activity/VWF antigen ratio (ADAMTS13:Act/VWF: Ag). This phenotype is mechanistically nonspecific and may reflect increased VWF release, impaired whole-molecule VWF clearance, reduced ADAMTS13 production, stability, or availability, functional inhibition of proteolysis, reduced VWF susceptibility to cleavage, or shared upstream inflammation. Human evidence from COVID-19 and cancer remains predominantly associative, whereas tumor-relevant perturbation studies provide preclinical causal support in defined models. The causal importance of the VWF–ADAMTS13 axis in human tumors nevertheless remains unproven. We therefore position this module within a broader thromboinflammatory network that includes complement activation, neutrophil extracellular traps, platelet activation, and tissue factor–thrombin–fibrin pathways. ICI-associated endothelial phenotypes are distinguished from rare ICI-associated immune TTP with categorical severe ADAMTS13 deficiency. Candidate interventions are accordingly presented as mechanistic research nodes requiring staged validation rather than as established oncology therapies.

## 1 Introduction

ADAMTS13 (a disintegrin and metalloproteinase with thrombospondin type 1 motifs, member 13) is best known as the enzyme whose severe deficiency causes thrombotic thrombocytopenic purpura (TTP) (1–3). By cleaving von Willebrand factor (VWF) under shear stress, ADAMTS13 limits the persistence and accumulation of ultra-large VWF (UL-VWF) multimers that drive platelet aggregation; severe loss of this activity defines TTP (1–3). This diagnostic framing, in which ADAMTS13 is viewed as either sufficient or severely deficient, has dominated clinical and research attention for two decades (1) and remains central to the differential diagnosis of thrombotic microangiopathies (4).

A broader functional role has emerged from studies of viral and inflammatory immunothrombosis, in which thrombus formation can serve as an effector arm of innate immunity (5). In severe COVID-19, sepsis, and chronic HIV infection, VWF antigen commonly rises while ADAMTS13 activity is normal or modestly reduced, and a low ADAMTS13/VWF ratio is associated with organ dysfunction or adverse outcomes (6–11). We use “relative ADAMTS13 insufficiency” as a proposed research phenotype for this load-to-capacity mismatch: ADAMTS13 activity remains outside the severe-deficiency range used for TTP, typically ≥10%, but is disproportionately low relative to the concurrent VWF burden. This phenotype does not, by itself, identify the underlying mechanism and should not be conflated with TTP or interpreted as evidence that ADAMTS13 is necessarily causal or rate limiting. In some vascular niches, it may reflect insufficient VWF-processing capacity relative to substrate burden, whereas in others it may primarily accompany endothelial activation and systemic inflammation.

Whether this proposed modifier has a causal role in human cancer remains unresolved. Many tumor microenvironments exhibit endothelial activation, complement dysregulation, platelet engagement, NET formation, and coagulation activation (12–15), yet evidence linking ADAMTS13 to malignancy remains fragmented across case reports, cohort associations, tissue-level observations, and a limited number of animal perturbation studies (16–21). Several alternative or complementary processes may contribute to the observed VWF–ADAMTS13 imbalance, including increased VWF secretion, impaired hepatic or splenic cellular clearance, altered ADAMTS13 synthesis, stability, or endocytosis, and parallel activation of inflammatory and coagulation pathways. These processes have rarely been assessed together in tumor settings. The purpose of this Hypothesis and Theory article is therefore to organize the available evidence into a bounded and falsifiable model, rather than to assume that ADAMTS13 insufficiency is the sole or dominant cause of cancer-associated thrombosis.

Here, we propose that the VWF–ADAMTS13 axis may function as a context-dependent thromboinflammatory modifier in selected tumor vascular niches. First, we define the molecular conditions under which this axis operates and distinguish ADAMTS13-mediated control of VWF multimer size from whole-molecule VWF clearance. Second, we examine evidence from viral and inflammatory settings while distinguishing human associations from perturbation-based evidence and considering alternative common-cause explanations. Third, we position the axis within the broader complement–NET–platelet–coagulation network and evaluate tumor- and ICI-related findings according to explicit evidence tiers. Finally, we define a measurable research phenotype, formulate falsifiable predictions, and outline the studies required before biomarker-guided intervention can be considered. **Figure 1** summarizes this framework.

**Figure 1:**
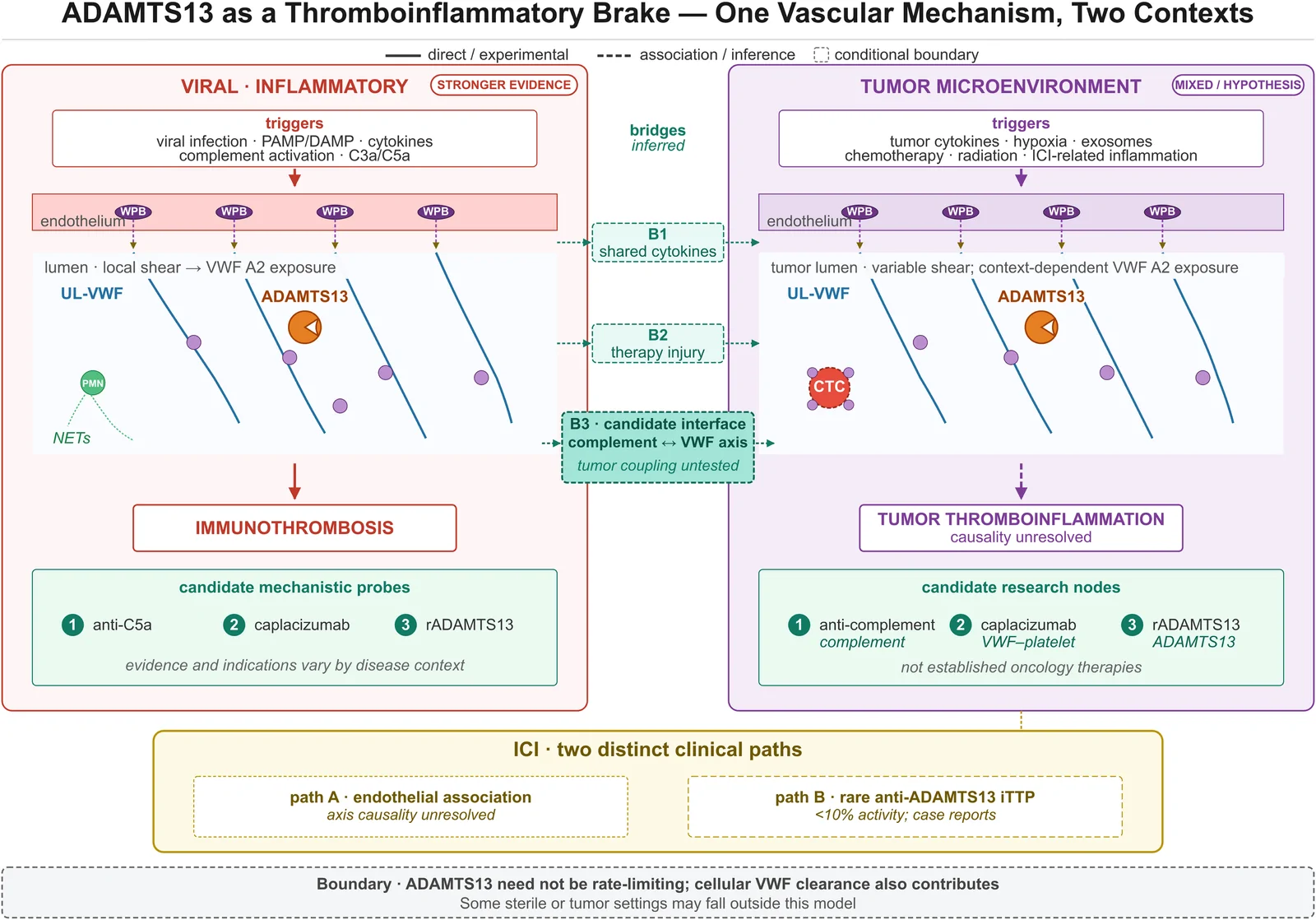
ADAMTS13 as a context-dependent thromboinflammatory brake across viral/inflammatory and tumor contexts. Both panels depict endothelial Weibel–Palade body exocytosis, endothelial-anchored UL-VWF strings, platelet recruitment, and proteolytic processing by ADAMTS13. The left panel summarizes viral and inflammatory evidence, whereas the right panel extends the model to tumor-associated vascular inflammation. Local force can expose the VWF A2 region; in heterogeneous tumor flow, A2 exposure is context dependent. The schematic emphasizes endothelial-anchored UL-VWF, while circulating and collagen-bound VWF and hepatic, splenic, and sinusoidal cellular clearance are discussed in Section 2.1. The boundary band indicates that cellular clearance also contributes and that ADAMTS13 need not be rate limiting. Solid lines denote direct or experimental support, dashed lines association or inference, and the outlined band a conditional boundary. The candidate complement–VWF interface is supported mainly by non-cancer evidence and remains untested in tumor vessels. The ICI box presents two distinct observations in the tumor-treatment context: an unresolved endothelial association and rare anti-ADAMTS13 immune TTP with severe categorical deficiency. The lower rows identify candidate mechanistic probes or research nodes; the evidence and clinical applicability vary by disease context, and none is established for cancer-associated relative insufficiency.

## 2 ADAMTS13 as a thromboinflammatory brake: molecular model and evidence from viral and inflammatory immunothrombosis

ADAMTS13 was identified as the protease deficient in TTP, and the dominant clinical paradigm has focused on its severe deficiency in acute TTP presentations. Recent work in viral immunothrombosis and severe inflammatory states, however, reveals a broader functional role that operates at activity levels well above the TTP diagnostic threshold. Here, we first describe the molecular operating model of ADAMTS13 as a thromboinflammatory brake at the vascular-immune interface, and then synthesize the multi-layered evidence from viral and inflammatory contexts that provides the empirical basis for this brake function.

### 2.1 Operating model at the vascular-immune interface

The core VWF–ADAMTS13 proteolytic circuit comprises four interacting elements. First, VWF is synthesized by endothelial cells and stored with P-selectin and other mediators in Weibel–Palade bodies as ultra-large multimers (22). Second, endothelial activation triggers luminal UL-VWF strings, while circulating plasma VWF provides a second substrate pool. Following endothelial disruption, exposed subendothelial collagen immobilizes circulating VWF, allowing it to bridge the damaged vessel wall to platelet GPIbα; surface anchorage and local hydrodynamic forces can extend VWF and increase platelet capture (23). Third, shear and tensile force act primarily on tethered VWF, unfolding its A2 domain and exposing the Tyr1605–Met1606 bond. VWF engagement through ADAMTS13 exosites promotes productive proteolysis, so VWF functions as both substrate and activating cofactor (24–28). Fourth, ADAMTS13 cleavage shortens platelet-reactive VWF multimers and reduces the VWF-dependent scaffold for platelet and leukocyte recruitment.

ADAMTS13-mediated proteolysis should be distinguished from whole-molecule clearance of VWF. Intact VWF and VWF–FVIII complexes are also removed by hepatic and splenic macrophages and sinusoidal endothelial cells, with contributions from receptors including CLEC4M, stabilin-2, and SCARA5 (29–31). Conversely, ADAMTS13 itself can undergo receptor-mediated uptake, including through Siglec-5/14 (32). Recent work further indicates that hepatic macrophage removal of ADAMTS13-cleaved VWF can be required for full protection *in vivo*, emphasizing complementarity rather than competition between cleavage and clearance (33). Tumor-associated myeloid and endothelial remodeling could alter VWF burden, ADAMTS13 availability, or both, but direct evidence in tumor-bearing hosts is lacking. The model therefore does not assume that ADAMTS13 is invariably rate limiting.

Within this framework, ADAMTS13 is positioned as a context-dependent modifier of VWF-dependent thromboinflammation. It acts downstream of cytokine release and endothelial activation by proteolytically reducing the multimeric size and platelet-adhesive potential of shear-exposed VWF (24–28). It does not suppress upstream inflammation, neutralize tissue factor or thrombin, dismantle NETs, or inhibit the entire complement cascade. We therefore use “thromboinflammatory brake” as a positional metaphor for this selective VWF-processing function, rather than to imply that ADAMTS13 is a master regulator or a universally rate-limiting determinant of thrombosis.

Relative ADAMTS13 insufficiency is used here as a proposed research phenotype, not as a clinical diagnosis, and should not be conflated with TTP. Operationally, it denotes ADAMTS13 activity outside the severe-deficiency range used for TTP, typically ≥10% of normal, together with increased VWF burden and a disproportionately low ADAMTS13:Act/VWF: Ag ratio (1–4, 34). ADAMTS13 activity, VWF: Ag, and the ratio should be reported separately, using a consistent ratio direction and values normalized to comparable reference-plasma standards (34). Activity below 10%, particularly in a compatible clinical syndrome, should instead prompt evaluation for categorical severe ADAMTS13 deficiency and immune-mediated or congenital TTP (1–4, 34).

No universal threshold has been validated for relative ADAMTS13 insufficiency across inflammatory diseases or cancer types. The ratio should therefore be analyzed primarily as a continuous variable and supplemented by serial change from a pretreatment baseline. If dichotomization is used, the cut-point should be prespecified, assay-specific, and externally validated; the CATS lower-quartile value of <0.31 should be regarded as an exploratory cohort benchmark rather than a clinical threshold (18). For discovery studies, one possible operational approach would be to combine VWF: Ag above a laboratory-specific upper reference limit, or a substantial rise from baseline, with a prespecified low ratio while retaining each component separately.

The ratio should be regarded as a pragmatic load-to-capacity index rather than a direct kinetic measurement. VWF: Ag primarily reflects circulating VWF protein abundance and does not directly characterize its multimeric distribution or platelet-binding function; platelet-dependent VWF activity, collagen-binding activity, or multimer analysis should therefore be added when feasible (35). Most clinical ADAMTS13 activity assays use peptide substrates under static conditions and may not reproduce the processing of native or anchored VWF under disease-relevant flow and shear conditions (34).Serial samples should ideally be analyzed on the same platform, and unexpected results should be confirmed with an alternative assay where feasible. Interpretation should also account for ABO blood group, age, acute-phase activity, liver dysfunction, hemolysis or hyperbilirubinemia, and recent plasma-containing therapy. Unexpectedly low ADAMTS13 activity should prompt measurement of ADAMTS13 antigen and inhibitor/IgG testing.

### 2.2 Evidence for a context-dependent ADAMTS13 brake in viral and inflammatory immunothrombosis

Evidence supporting ADAMTS13 as a thromboinflammatory modifier beyond its established role in TTP can be organized into five evidence domains: observational evidence of an inflammation-associated VWF–ADAMTS13 imbalance, clinical endpoint associations in viral immunothrombosis, candidate molecular mechanisms, upstream pharmacological observations, and genetic evidence in non-viral immunothrombosis. These domains differ substantially in causal strength and should therefore be interpreted separately.

Observational basis for inflammation-associated relative insufficiency. Bockmeyer et al. reported a prospective longitudinal cohort encompassing systemic inflammation after extracorporeal cardiopulmonary circulation and severe sepsis. ADAMTS13 activity declined stepwise with the severity of the inflammatory response, accompanied by the appearance of plasma UL-VWF multimers, and these changes were associated with organ dysfunction and lethality (6). These observations provide an empirical basis for considering a relative VWF–ADAMTS13 imbalance in inflammatory states, but do not establish that inflammation directly causes the decline in ADAMTS13 activity or that ADAMTS13 becomes rate limiting. Sepsis-focused reviews describe a similar pattern across heterogeneous etiologies, with increased VWF burden and relatively reduced ADAMTS13 activity during inflammatory endothelial activation (7).

Clinical endpoint associations in COVID-19. In hospitalized patients with COVID-19, a combined pattern of elevated VWF antigen, lower ADAMTS13 activity, and complement activation was associated with mortality (10). This concurrence identifies a prognostic thromboinflammatory signature but does not establish a causal sequence among VWF, ADAMTS13, and complement. These abnormalities may instead arise in parallel from common upstream processes, including endothelial injury and systemic inflammation. Similarly, reduced ADAMTS13/VWF ratios together with markers of endothelial injury in severe disease are consistent with a load-to-capacity imbalance, but human observational data cannot determine whether ADAMTS13 dysfunction is a driver, an amplifier, or predominantly a biomarker.

Potential mechanisms of reduced effective VWF-processing capacity. Why measured ADAMTS13 activity or effective VWF-processing capacity decreases during inflammation remains unresolved and is likely context dependent. Several non-mutually exclusive mechanisms may contribute. Inflammatory cytokines may alter ADAMTS13 expression (36), while IL-6 can inhibit cleavage of endothelial UL-VWF strings under flow (37). Neutrophil-derived oxidants can directly impair ADAMTS13 proteolytic activity (38), whereas oxidative modification of the VWF cleavage region may reduce substrate susceptibility to ADAMTS13 (39). Receptor-mediated endocytosis, including uptake through Siglec-5/14, may further reduce circulating ADAMTS13 availability (32). In addition, a large acute release of VWF may generate a kinetic substrate–protease mismatch even without a marked absolute reduction in ADAMTS13. S-protein-associated NLRP3/IL-1β activation accompanied by increased VWF without compensatory ADAMTS13 upregulation, together with CFH-variant effects on the VWF–ADAMTS13 axis, illustrate possible regulatory inputs (40, 41), but neither explains why ADAMTS13 activity is reduced in tumor microenvironments. Most of these candidate mechanisms have been demonstrated *in vitro*, ex vivo, or outside cancer and therefore require direct testing in tumor-bearing systems.

Upstream pharmacological observation. In severe COVID-19, anti-C5a therapy was associated with attenuation of the decline in ADAMTS13 activity relative to standard care (42). This finding is compatible with an effect of upstream complement or endothelial regulation on the measured VWF–ADAMTS13 axis, but the biomarker change does not establish that complement directly caused ADAMTS13 loss or that restored VWF processing mediated clinical benefit. It therefore supports mechanistic plausibility and experimental prioritization rather than a completed causal pathway.

Genetic and leukocyte-anchored evidence beyond acute viral immunothrombosis. Stronger perturbational evidence comes from non-viral inflammatory models. In a *Staphylococcus aureus* sepsis model, Peetermans et al. showed that *vwf*-deficient mice had reduced mortality and enhanced bacterial clearance, whereas *adamts13*-deficient mice had increased mortality, with these differences largely attributable to differences in organ microthrombus burden (43). This genetic contrast provides preclinical evidence that altering the VWF–ADAMTS13 circuit can modify thromboinflammatory outcomes in a defined inflammatory context. Conceptually, it parallels the zebrafish tumor model discussed later, in which loss of ADAMTS13 worsens outcome whereas removal of the VWF substrate is protective.

Consistent with the involvement of a broader leukocyte–vascular network, virally suppressed HIV infection is accompanied by persistent complement dysregulation, altered VWF–ADAMTS13 dynamics, increased neutrophil extracellular trap (NET) formation, and platelet activation, while C5a receptor antagonism reduces NET formation (11). NETs themselves provide a scaffold for VWF-dependent thrombosis and contribute causally to deep-vein thrombosis in mouse models (44, 45). These observations support a leukocyte-anchored amplification pathway capable of interacting with VWF–platelet–ADAMTS13 imbalance, although the relative contribution of each component is likely to vary by disease context.

Taken together, these evidence domains support the biological plausibility of ADAMTS13 as a context-dependent thromboinflammatory modifier, but their causal strength is unequal. Genetic perturbation studies provide preclinical causal evidence in defined models, whereas human pharmacological and observational studies predominantly provide association or mechanistic plausibility. The key unresolved questions are whether ADAMTS13 becomes functionally limiting in particular tumor vascular niches, whether a low ADAMTS13/VWF phenotype precedes and contributes to thromboinflammatory events rather than simply tracking endothelial injury, and whether selective restoration of VWF-processing capacity alters VWF-dependent outcomes.

### 2.3 Evidence hierarchy, competing explanations, and falsifiability

To avoid conflating distinct types of evidence, we classify the supporting observations into three tiers. Established mechanism refers to replicated biochemical evidence or human disease evidence with direct causal support, exemplified by severe ADAMTS13 deficiency in TTP and shear-dependent cleavage of VWF (1–3, 24–28). Emerging evidence includes perturbational animal studies, disease-relevant tissue observations, and prospective clinical associations (18–21, 43); within this tier, preclinical causal evidence is distinguished explicitly from human associative evidence. Hypothesis-generating extension refers to extrapolation across disease contexts, including application of the brake model to selected tumor vascular niches, a proposed reciprocal complement–VWF–ADAMTS13 interface within tumor vessels, a putative ICI-associated relative phenotype, and therapeutic application of this axis in oncology. The biological observations and pharmacological precedents motivating these extensions are discussed below (19, 41, 46–52), but the extensions themselves remain unproven. Prospective clinical association alone is not considered mechanistic proof.

A high-VWF/low-ADAMTS13 phenotype can arise through several competing or complementary mechanisms, including increased endothelial VWF release (21, 53, 54), reduced hepatic or splenic VWF clearance (29–31, 33), altered ADAMTS13 synthesis or availability (32, 36), functional inhibition of ADAMTS13 or its cleavage reaction (37, 38), reduced susceptibility of VWF to cleavage (39), or a shared upstream inflammatory process. These mechanisms are not mutually exclusive, and most have not been examined simultaneously in tumor-bearing systems. The causal-modifier hypothesis would be weakened if selective perturbation of ADAMTS13 fails to alter VWF-dependent thrombus formation after upstream endothelial injury and major competing pathways are controlled, if the effect persists after removal of VWF dependence, or if the ADAMTS13:Act/VWF: Ag ratio provides no temporal or mechanistic information beyond established markers of endothelial injury and parallel thromboinflammatory pathways. Conversely, evidence that ADAMTS13 loss and restoration produce reciprocal, VWF-dependent effects under defined vascular conditions would strengthen the hypothesis.

## 3 Mechanistic bridges from viral immunothrombosis to the tumor microenvironment

Phenomenological convergence, with many thromboinflammatory viral states and selected tumor microenvironments showing VWF elevation, complement activation, and platelet engagement, does not by itself establish mechanistic continuity. The relevant question is whether shared cellular processes or molecular interfaces connect these settings. Three such points of continuity are particularly important: shared endothelial activation driving WPB exocytosis, therapy-induced endothelial injury, and a reciprocal molecular interface between complement and the VWF-ADAMTS13 axis. We address each in turn, then define the conditions under which the brake model applies and those under which it does not.

### 3.1 Shared endothelial activation and Weibel-Palade body exocytosis

The first point of continuity lies in endothelial activation that drives WPB exocytosis and UL-VWF release. At rest, the endothelium maintains a nonthrombogenic surface; upon activation by pro-inflammatory mediators, it becomes an active participant in the inflammatory response (55, 56). WPBs co-store UL-VWF, P-selectin, and the chemokine IL-8, and VWF itself directs IL-8 packaging into these organelles (57). A single exocytic event can therefore release both an adhesive substrate and a chemotactic signal. Consistently, inflammation is an established determinant of circulating VWF levels (53).

This activation pathway is not restricted to viral infection or sterile inflammation. Tumor-derived soluble factors can also directly activate macrovascular and microvascular endothelium. Goerge et al. showed that melanoma and colon cancer cell supernatants induce endothelial calcium flux and secretion of VWF and IL-8 through a matrix metalloproteinase-1/protease-activated receptor-1 axis (54). Bauer et al. further demonstrated in malignant melanoma that tumor cells activate endothelial cells, leading to luminal release of VWF fibers and platelet aggregation in tumor microvessels. Human and murine melanoma tissues also showed increased VWF release together with local reduction of ADAMTS13 proteolytic activity and protein expression (21). These findings indicate that at least some tumor microenvironments can reproduce the endothelial-activation-to-UL-VWF-release sequence observed in inflammatory immunothrombosis. The remaining uncertainty is mainly quantitative and context-specific: formal comparisons of WPB exocytosis kinetics across viral, septic, and tumor stimuli have not, to our knowledge, been performed.

### 3.2 Therapy-induced endothelial injury

Therapy-induced endothelial injury provides another point of continuity. Across several major treatment modalities, cancer therapy can impose inflammatory and prothrombotic stress on the endothelium, although the degree of mechanistic resolution differs by modality. Anthracycline chemotherapy induces a procoagulant endothelial phenotype, increasing tissue factor activity, platelet-endothelial adhesion, and microthrombus formation (58–60). Ionizing radiation increases endothelial VWF release through enhanced transcription and produces microvascular inflammation and microthrombi in irradiated tissue (61–63). Immune checkpoint inhibitors are associated with accelerated atherosclerotic plaque progression and a higher rate of cardiovascular events (64), indicating an inflammatory vascular effect operating alongside antitumor action.

Zheng et al. used an *in vivo* zebrafish model combining ionizing radiation with melanoma transplantation, thereby placing the VWF-ADAMTS13 axis within a tumor-bearing and therapy-stressed vascular context (20). adamts13-null fish developed accelerated thrombocytopenia and earlier mortality than wild-type controls, whereas vwf-null fish were partially protected, supporting substrate dependence of the deleterious adamts13-null phenotype. This model is important because it places ADAMTS13 loss directly within a tumor-bearing, therapy-stressed vascular context, rather than relying solely on extrapolation from inflammatory or viral settings. Transcriptomic profiling of tumor explants from adamts13-deficient animals further revealed altered oxidation-reduction, lipid metabolism, and steroid synthesis pathways (20) suggesting broader microenvironmental alterations beyond thrombocytopenia alone.

### 3.3 A reciprocal molecular interface between complement and the VWF-ADAMTS13 axis

The third point of continuity provides the most direct mechanistic link in the proposed model. The evidence does not support a simple one-directional hierarchy in which complement acts upstream, injures the endothelium, and secondarily induces VWF release. Instead, it supports a reciprocal molecular interface between complement and the VWF-ADAMTS13 axis, with each system capable of modulating the other.

In one direction, complement proteins modulate VWF–ADAMTS13 mechanics. Cao et al. showed that the atypical hemolytic uremic syndrome-associated complement factor H variant W1183R-CFH, also designated p.Trp1183Arg, enhanced ADAMTS13-mediated cleavage of VWF under shear stress, whereas wild-type CFH did not show this effect despite comparable binding affinity of both proteins for VWF (41). This effect was attributed to W1183R-CFH-specific conformational changes in the VWF A2 domain, which increased accessibility of the Tyr1605–Met1606 scissile bond (41). On this evidence, complement proteins may influence not only VWF substrate availability but also the efficiency of ADAMTS13-mediated VWF proteolysis.

In the reverse direction, the VWF-ADAMTS13 axis also modulates complement. VWF acts as a cofactor for factor I-mediated cleavage of C3b, and this regulatory function depends on VWF multimer size: smaller VWF multimers promote C3b inactivation, whereas ultra-large VWF multimers more readily permit spontaneous complement activation (46). Consistently, endothelial-anchored UL-VWF strings can serve as assembly platforms for alternative-pathway complement components (47). Tati et al. further showed in human and murine thrombotic microangiopathy that ADAMTS13 deficiency promotes complement activation on VWF-platelet strings through the alternative pathway (48). The functional implication is that ADAMTS13 cleavage of UL-VWF not only limits platelet adhesion but also restrains complement amplification, thereby linking the brake directly to complement control.

This reciprocal interface has so far been characterized mainly in aHUS, TTP, and *in vitro* shear systems. Its relevance to the tumor microenvironment remains a hypothesis rather than an established fact, but the necessary biological substrates appear to be present. The tumor microenvironment displays extensive complement dysregulation, including C5a-driven immunosuppression (49, 65). We therefore propose, as a testable hypothesis, that tumor-associated complement dysregulation and the VWF-ADAMTS13 axis may interact through this same reciprocal interface. In this setting, brake failure may both permit local complement activation and be further reinforced by it. Whether this loop operates in tumor vessels has not, to our knowledge, been directly tested. The reciprocal complement–VWF-ADAMTS13 interface proposed here therefore provides a mechanistic hypothesis for future experimental studies.

### 3.4 The VWF–ADAMTS13 module within the broader thromboinflammatory network

The VWF–ADAMTS13 axis represents one adhesive, shear-sensitive module within a broader thromboinflammatory network rather than a master regulator. Complement activation can promote endothelial injury and assemble on endothelial UL-VWF strings, while NETs provide extracellular scaffolds that support platelet adhesion and coagulation (41, 44–48). Activated platelets further recruit leukocytes and provide procoagulant surfaces, whereas tumor-associated tissue factor, factor Xa, and thrombin promote fibrin formation and can amplify endothelial activation (12–15, 66–68). These pathways can intersect with and reinforce VWF-dependent thromboinflammation, but each can also contribute to thrombosis through mechanisms that do not require impaired ADAMTS13 activity. Accordingly, ADAMTS13 should be viewed as selectively regulating a VWF-dependent platelet–leukocyte adhesive platform rather than directly suppressing tissue factor–thrombin signaling, dismantling NETs, or broadly inhibiting complement.

The modifier hypothesis is therefore expected to be most relevant in vascular settings characterized by abundant platelet-reactive VWF, sufficient local force to expose the VWF A2 domain, and substantial dependence on VWF-mediated platelet recruitment (25–28). By contrast, complement-dominant, NET-dominant, or tissue factor–thrombin–fibrin-dominant lesions may develop despite preserved ADAMTS13 activity (41, 44–48, 66–68). Cancer-associated thrombosis is mechanistically heterogeneous and frequently involves strong coagulation- and fibrin-dependent components (12–15, 66–68); therefore, associations of the VWF–ADAMTS13 axis with overall VTE risk or survival should be regarded as clinically relevant but mechanistically nonspecific (18, 19). More informative tests of the proposed modifier would focus on VWF-rich microvascular phenotypes and determine whether selective manipulation of ADAMTS13 alters VWF-dependent platelet recruitment or thrombus formation after competing pathways are accounted for.

### 3.5 Boundary conditions: shear, conformation, and rate limitation

The conditional nature of the ADAMTS13 “brake” reflects both the mechanical state of the VWF substrate and the conformational regulation of ADAMTS13. Under low hydrodynamic force, VWF remains relatively compact and its A2 cleavage region is poorly accessible (23, 27). Circulating ADAMTS13, meanwhile, predominantly adopts a closed conformation (25, 28); this state should not be interpreted as being directly controlled by shear. When VWF is immobilized on collagen or engaged by platelets, local tensile forces can extend the multimer and expose the Tyr1605–Met1606 bond within the A2 domain (23, 25–27). Subsequent engagement of VWF with ADAMTS13 exosites can allosterically promote a proteolytically competent conformation and facilitate productive cleavage (25, 26, 28). Thus, mechanical force acts primarily on the VWF substrate, whereas ligand-dependent activation regulates ADAMTS13 activity. This division of roles provides a mechanistic basis for why the proposed brake is context dependent rather than constitutively engaged.

The model also has biological boundaries. In a sterile peripheral-ischemia model, ADAMTS13 deficiency did not alter arteriogenesis or angiogenesis (69), indicating that loss of ADAMTS13 does not necessarily modify every form of vascular remodeling. This finding should be regarded as a model-specific boundary rather than evidence that ADAMTS13 is irrelevant to sterile vascular processes more generally. Accordingly, the modifier hypothesis is expected to be most applicable where platelet-reactive VWF is abundant, local mechanical forces permit A2 exposure, and thrombosis depends substantially on VWF-mediated platelet recruitment. In settings dominated by alternative thromboinflammatory or remodeling pathways, ADAMTS13 may remain functionally non-limiting.

## 4 ADAMTS13 in the tumor microenvironment and immune checkpoint blockade

The relevance of the ADAMTS13-VWF brake in cancer is expected to depend on the degree of thromboinflammatory pressure generated by the tumor microenvironment. We therefore assess the available tumor evidence and then examine immune checkpoint blockade as a clinical setting in which endothelial activation, immune dysregulation, and ADAMTS13 insufficiency may converge.

### 4.1 ADAMTS13 in the tumor microenvironment

Cancer-associated thrombosis is a major clinical burden, and solid tumors often contain structurally abnormal and heterogeneously perfused vasculature (13–15, 70). Tumor-derived tissue factor-bearing extracellular vesicles, factor Xa, matrix metalloproteinase-1, and inflammatory mediators can activate endothelial signaling, including PAR-1-dependent pathways, and promote the release of VWF and IL-8 (54, 66–68). These conditions may increase the local availability and persistence of platelet-reactive VWF. However, VWF abundance is determined not only by endothelial release but also by whole-molecule clearance through hepatic and splenic macrophages and sinusoidal endothelial cells (29–31, 33). Whether these clearance pathways are altered in tumor-bearing hosts remains poorly characterized. Accordingly, the dominant determinant of a VWF-high state—enhanced release, impaired cellular clearance, insufficient proteolytic processing, or parallel coagulation-driven mechanisms—may differ across tumor contexts and remains unresolved.

Human evidence linking the VWF–ADAMTS13 axis to cancer-associated thrombosis remains substantially less definitive than the established causal biology of severe ADAMTS13 deficiency in TTP. In the 795-patient Vienna Cancer and Thrombosis Study, higher VWF levels and a lower ADAMTS13 activity/VWF antigen ratio were associated with venous thromboembolism, whereas ADAMTS13 activity alone was not (18). The ratio may therefore reflect the balance between VWF burden and ADAMTS13 activity, but it does not distinguish whether the observed imbalance is driven primarily by increased VWF, reduced ADAMTS13, altered clearance, or a shared upstream inflammatory process. Nor does the association establish that ADAMTS13 is independently causal or rate limiting. The cohort-derived lower-quartile value of <0.31 can be used as an exploratory benchmark for research stratification, but it should not be regarded as a validated diagnostic or treatment threshold (18).

Second, Zheng et al. provided the most direct mechanistic evidence in a zebrafish model combining ionizing radiation with melanoma transplantation: adamts13-null fish developed accelerated thrombocytopenia and earlier mortality than wild-type controls, whereas vwf-null fish were partially protected, indicating VWF substrate dependence of the deleterious adamts13-null phenotype (20). Transcriptomic profiling of tumor explants from adamts13-deficient animals further revealed altered oxidation-reduction, lipid metabolism, and steroid synthesis pathways, suggesting that ADAMTS13 deficiency in a tumor-bearing context is associated with microenvironmental alterations beyond thrombocytopenia alone (20).

Third, Bauer et al. provided disease-relevant tissue-level evidence in malignant melanoma. Both human and murine melanoma tissues displayed increased luminal VWF release together with local reduction of ADAMTS13 proteolytic activity and protein expression (21). This finding supports the presence of VWF-ADAMTS13 brake imbalance within intact tumor vasculature, rather than only indirect inference from experimental models.

Fourth, Stadler et al. reported in a prospective cohort of patients with metastatic melanoma treated with immune checkpoint inhibitors that baseline plasma VWF antigen was associated with progression-free and overall survival, whereas ADAMTS13 activity was associated with overall survival in univariate analysis (19). Responders and patients with resistant disease also showed divergent VWF dynamic trajectories. This cohort-level evidence indicates that the VWF-ADAMTS13 axis carries prognostic information at the population level in a contemporary immuno-oncology setting.

Taken together, the available tumor evidence is strongest in melanoma but remains insufficient to establish causality in humans. The modifier hypothesis therefore generates several falsifiable predictions. After upstream endothelial activation and major competing pathways are controlled, selective loss of ADAMTS13 should increase the persistence of tumor-vessel UL-VWF and VWF-dependent platelet-rich microthrombi. Removal of VWF or blockade of the VWF–GPIb interaction should substantially attenuate or abolish this effect, whereas restoration of ADAMTS13 activity should reduce VWF-dependent thrombus burden without necessarily altering upstream inflammatory signaling. In prospective human studies, a low ADAMTS13:Act/VWF: Ag phenotype should demonstrate temporal precedence and provide information beyond tumor burden, systemic inflammation, complement activation, NET formation, platelet activation, and tissue factor–thrombin markers. Until such temporal, mechanistic, and intervention-based evidence is obtained, associations with VTE or survival should be regarded as clinically relevant but mechanistically nonspecific.

### 4.2 Immune checkpoint blockade: two distinct observations

Current evidence surrounding ICI therapy contains two distinct VWF–ADAMTS13-related observations that should not be conflated: a VWF-high endothelial phenotype observed in some patients during ICI treatment and rare immune TTP with categorical severe ADAMTS13 deficiency (17, 19, 71, 72). The first represents an associative vascular phenotype that may reflect tumor activity, treatment response, systemic inflammation, or other shared upstream influences; the second represents an abrupt autoimmune loss of ADAMTS13 activity. Neither observation currently supports a common ICI-specific mechanism of relative ADAMTS13 insufficiency.

The first observation concerns vascular and endothelial abnormalities documented in patients receiving ICI therapy. ICI exposure has been associated with cardiovascular and thromboembolic events (64, 73), but available studies do not separate a direct drug effect on VWF or ADAMTS13 from the effects of tumor burden, treatment response, comorbid disease, or systemic inflammation. In a prospective melanoma cohort sampled before and during ICI therapy, ADAMTS13 activity did not change significantly over time, whereas longitudinal VWF trajectories were associated with treatment response (19). In the absence of a non-ICI or alternative-treatment cancer comparator, these findings cannot be attributed specifically to checkpoint blockade and should therefore be regarded as emerging human associative evidence rather than evidence of a direct ICI–VWF–ADAMTS13 mechanism.

The second observation is rare ICI-associated immune TTP. Case reports describe severe, often inhibitor-mediated ADAMTS13 deficiency associated with anti-ADAMTS13 autoimmunity after checkpoint blockade (17, 71). This represents a categorical failure of the protease, typically with activity <10%, rather than the continuous VWF-high/low-ratio phenotype proposed here. It therefore should not be used as evidence that relative ADAMTS13 insufficiency explains vascular events in most ICI-treated patients. Moreover, ICI-associated thrombotic microangiopathy has also been reported with preserved ADAMTS13 activity, emphasizing that complement-dominant and other endothelial-injury mechanisms must remain in the differential diagnosis (72).

These two observations require different research and clinical approaches. Longitudinal studies of the putative endothelial phenotype should incorporate pretreatment sampling, non-ICI or alternative-treatment cancer comparators where feasible, adjustment for tumor response and inflammatory burden, and parallel assessment of endothelial, complement, NET, platelet, and coagulation markers. By contrast, suspected immune TTP requires urgent diagnostic evaluation and treatment according to established pathways for severe ADAMTS13 deficiency (1–4). The rare autoimmune syndrome is therefore best regarded as a clinically important categorical boundary condition rather than mechanistic support for a continuous ICI-associated ADAMTS13:Act/VWF: Ag phenotype.

## 5 Translational implications and validation roadmap

This framework does not imply an automatic treatment recommendation. Instead, it distinguishes three mechanistic patterns: categorical severe ADAMTS13 failure, relative VWF-processing imbalance, and complement-dominant thrombotic microangiopathy. It also defines the evidence needed before mechanism-matched interventions can be tested in oncology.

### 5.1 Three mechanistic patterns in oncology-associated thromboinflammation

Oncology-associated thromboinflammation spans at least three overlapping patterns. Categorical ADAMTS13 failure is exemplified by immune TTP, with activity typically <10% and often an inhibitor (1–4). Relative VWF-processing imbalance is a proposed research phenotype with activity outside that severe range but disproportionate to elevated VWF burden. Complement-dominant TMA may occur with preserved ADAMTS13 activity, as reported after ICI or selected chemotherapies (72, 74, 75). These patterns require clinical adjudication rather than assignment by a single biomarker.

The patterns can interact but are not interchangeable. Complement activation can amplify endothelial injury and assemble on VWF-rich surfaces, whereas ADAMTS13 cleavage can limit one VWF-dependent platform for complement and platelet recruitment (41, 46–48). This coupling does not make all complement-dominant disease an ADAMTS13 disorder, nor does a low ratio establish that ADAMTS13 replacement is appropriate. **Table 1** summarizes the distinctions and unresolved tests.

**Table 1:** Operational distinction among categorical ADAMTS13 failure, relative VWF-processing imbalance, and complement-dominant thrombotic microangiopathy.

Feature	Categorical ADAMTS13 failure	Relative VWF-processing imbalance	Complement-dominant TMA
Working definition	Severe ADAMTS13 activity loss, typically <10%, often inhibitor-mediated	ADAMTS13 activity outside the severe range with elevated VWF and low ADAMTS13:Act/VWF: Ag	TMA phenotype with preserved ADAMTS13 and evidence of complement dysregulation
Status	Established disease mechanism in TTP	Proposed research phenotype; not a diagnosis	Clinical/mechanistic category requiring adjudication
Representative oncology context	Rare ICI-associated immune TTP	VWF-high tumor or inflammatory vascular niches	Selected ICI- or chemotherapy-associated TMA
Causal interpretation	Direct causal failure when compatible TTP syndrome is present	Driver, amplifier, or biomarker remains to be determined	Complement may dominate; ADAMTS13 is not necessarily causal
Minimum measurements	ADAMTS13 activity, antigen, inhibitor/IgG; hemolysis and organ injury	ADAMTS13 activity and antigen; VWF: Ag and activity; ratio; serial change; multimers where feasible	ADAMTS13 plus complement activation, hemolysis, kidney injury, and alternative TMA evaluation
Competing explanations	Congenital deficiency; other causes of severe acquired loss	VWF release or impaired clearance; altered ADAMTS13 production, stability, uptake, or substrate susceptibility	Drug toxicity, malignant hypertension, infection, transplantation, or other endothelial injury
Evidence needed in cancer	Case confirmation and pharmacovigilance	Temporal cohorts; tumor-flow perturbation; VWF epistasis; ADAMTS13 rescue	Prospective phenotyping and complement target-engagement studies
Candidate research node	Established TTP treatment pathway	ADAMTS13 replacement or VWF blockade only after phenotype validation	Complement inhibition only in selected validated settings
Key safety issue	Delay in urgent TTP treatment	Bleeding or immunogenicity; interaction with thrombocytopenia/anticoagulation	Serious infection and diagnostic misclassification

Relative ADAMTS13 insufficiency is a hypothesis-generating research phenotype and has no validated universal diagnostic threshold. ADAMTS13 activity <10% in a compatible syndrome should prompt urgent evaluation for TTP. Treatment statements indicate research or established disease-specific pathways, not recommendations for cancer-associated thrombosis.

ADAMTS13, a disintegrin and metalloproteinase with thrombospondin type 1 motifs, member 13; ICI, immune checkpoint inhibitor; NET, neutrophil extracellular trap; TMA, thrombotic microangiopathy; TTP, thrombotic thrombocytopenic purpura; VWF, von Willebrand factor; VWF: Ag, VWF antigen.

### 5.2 Candidate intervention nodes and validation requirements

The following interventions are considered as candidate mechanistic research nodes rather than established therapies for cancer-associated relative VWF-processing imbalance. Recombinant ADAMTS13 (apadamtase alfa) restored ADAMTS13 activity and demonstrated favorable clinical and pharmacodynamic outcomes in a phase 3 trial in congenital TTP (50) and is approved for congenital TTP (51). These findings establish the feasibility of restoring ADAMTS13 activity in severe congenital deficiency, but they do not demonstrate efficacy in immune TTP, cancer-associated thrombosis, or ICI-associated vascular inflammation. Before recombinant ADAMTS13 could be evaluated for the proposed oncology phenotype, studies would need to demonstrate that platelet-reactive, cleavage-susceptible VWF persists because ADAMTS13-mediated proteolytic capacity is functionally limiting. Safety assessment should include anti-ADAMTS13 antibodies and neutralizing responses (76). Infusion-related reactions and bleeding-related interactions with thrombocytopenia, anticoagulation, or tumor-associated hemorrhagic risk should also be considered.

Caplacizumab inhibits the interaction between VWF and platelet GPIb and has randomized clinical evidence in acquired immune TTP (77). However, it acts downstream of VWF availability and does not restore ADAMTS13 activity, and its efficacy has not been established in cancer-associated relative VWF-processing imbalance. Any oncology-directed investigation would first require evidence that the relevant vascular phenotype is substantially VWF-dependent. Early clinical studies would also require careful assessment of bleeding, particularly in patients with thrombocytopenia, concurrent anticoagulation, mucosal tumors, or treatment-related coagulopathy (77).

Complement inhibition represents a mechanistically distinct intervention. Although complement-directed therapy is biologically plausible in selected complement-mediated disorders, evidence in cancer therapy-associated TMA remains limited and is derived largely from case reports or small clinical series (72, 74, 75). Similarly, the association between anti-C5a treatment and attenuation of the decline in ADAMTS13 activity in severe COVID-19 (42) is hypothesis-generating; it does not establish that complement inhibition restores ADAMTS13 function or that changes in VWF processing mediate clinical benefit. Oncology studies of complement-directed therapy should therefore demonstrate complement target engagement, define whether complement is functionally dominant rather than merely associated with endothelial injury, and incorporate appropriate infection and other safety monitoring.

Translation should proceed through a staged validation pathway. First, prospective multicenter cohorts should obtain pretreatment and serial samples and measure ADAMTS13 activity and antigen, inhibitor/IgG status, VWF: Ag, VWF platelet- or collagen-binding activity, VWF propeptide, and VWF multimers, together with markers of complement activation, NET formation, platelet activation, tissue factor–thrombin signaling, and endothelial injury. Clinical outcomes should include adjudicated VTE, TMA, bleeding, organ-specific microvascular injury, and tumor response. Second, tumor–endothelium flow systems and tumor-bearing animal models should test ADAMTS13 loss and gain of function, VWF epistasis, and recombinant ADAMTS13 rescue under defined mechanical conditions. Third, any biomarker-based enrichment strategy should be prespecified, externally validated, and shown to provide incremental information beyond tumor stage, systemic inflammation, D-dimer, treatment exposure, and competing thromboinflammatory pathways. Only after these steps should biomarker-enriched early-phase trials evaluate safety, pharmacodynamic target engagement, and preliminary efficacy of mechanism-informed interventions.

### 5.3 Conclusion

ADAMTS13 is an established regulator of shear-exposed VWF, and severe ADAMTS13 deficiency is a proven causal mechanism in TTP. Beyond TTP, the available evidence supports a more bounded model in which an imbalance between VWF burden and ADAMTS13 processing capacity may act as a context-dependent modifier or amplifier of thromboinflammation in selected VWF-rich and mechanically permissive tumor vascular niches. Tumor-relevant perturbation studies provide preclinical causal support in defined models, whereas human tumor-tissue observations and prospective cohort data remain associative.

The proposed relative VWF-processing imbalance is mechanistically nonspecific. It may arise from increased endothelial VWF release, impaired cellular VWF clearance, altered ADAMTS13 production, stability, or availability, reduced susceptibility of VWF to proteolysis, or shared upstream inflammatory processes. Whether ADAMTS13 becomes functionally limiting in human tumors, whether a complement–VWF interface contributes causally within tumor vessels, whether ICI therapy directly alters the VWF–ADAMTS13 axis, and whether biomarker-guided intervention can improve clinical outcomes all remain unresolved.

The value of this framework therefore lies not in establishing a new treatment paradigm, but in making the hypothesis testable. It separates established mechanisms from preclinical causal evidence, human association, and hypothesis-generating extension; defines competing explanations and boundary conditions; and identifies experiments capable of determining when ADAMTS13 functions as a driver, an amplifier, or primarily a biomarker of thromboinflammatory injury.

## Data Availability

The original contributions presented in the study are included in the article/supplementary material. Further inquiries can be directed to the corresponding author.
